# How shape-based anthropometry can complement traditional anthropometric techniques: a cross-sectional study

**DOI:** 10.1038/s41598-020-69099-4

**Published:** 2020-07-22

**Authors:** Michael Thelwell, Chuang-Yuan Chiu, Alice Bullas, John Hart, Jon Wheat, Simon Choppin

**Affiliations:** 1 Centre for Sports Engineering Research, Advanced Wellbeing Research Centre, Sheffield Hallam University, Sheffield, S9 3TU UK; 2 College of Health, Wellbeing and Life Sciences, Sheffield Hallam University, Sheffield, S10 2DN UK

**Keywords:** 3-D reconstruction, Epidemiology

## Abstract

Manual anthropometrics are used extensively in medical practice and epidemiological studies to assess an individual's health. However, traditional techniques reduce the complicated shape of human bodies to a series of simple size measurements and derived health indices, such as the body mass index (BMI), the waist-hip-ratio (WHR) and waist-by-height^0.5^ ratio (WHT.5R). Three-dimensional (3D) imaging systems capture detailed and accurate measures of external human form and have the potential to surpass traditional measures in health applications. The aim of this study was to investigate how shape measurement can complement existing anthropometric techniques in the assessment of human form. Geometric morphometric methods and principal components analysis were used to extract independent, scale-invariant features of torso shape from 3D scans of 43 male participants. Linear regression analyses were conducted to determine whether novel shape measures can complement anthropometric indices when estimating waist skinfold thickness measures. Anthropometric indices currently used in practice explained up to 52.2% of variance in waist skinfold thickness, while a combined regression model using WHT.5R and shape measures explained 76.5% of variation. Measures of body shape provide additional information regarding external human form and can complement traditional measures currently used in anthropometric practice to estimate central adiposity.

## Introduction

Measurements of size and shape of the human body are an important source of information for a range of scientific fields and applications. Traditional manual anthropometrics have been used extensively in medical practice and epidemiological studies to derive health risk indicators, since it has been suggested that human body shape is dependent on its underlying composition, including soft and skeletal tissues^1^. Indices, such as the body mass index (BMI), waist girth and the waist-hip ratio (WHR) are used to assess variations in human body dimensions and physical health^2–4^. Of these, BMI is most commonly used in current practice to determine the healthy weight range for individuals based on their height. However, BMI fails to distinguish between quantities of muscle and fat, which are of different density, and therefore is prone to misclassifying muscular individuals as being overweight or obese^5,6^. Size measures, such as sagittal diameter, waist girth and WHT.5R have been found to demonstrate improved correlations with quantities of abdominal visceral fat and greater associations with metabolic disease risks compared to BMI ^7,8^. Relative measures, such as the WHR, provide information about the size of the abdomen relative to the rest of the body, so has been used as a proxy of torso shape and central obesity, defined as excess fat around the abdominal region^7^. However, these relatively simple approaches to measuring external human form only utilise a small number of manual anthropometrics, which are prone to human error and limited by their simplicity, as they do not fully describe the complex three-dimensional (3D) variations in human body shape^4,9–11^. Skinfold thickness measurements are a manual anthropometric technique which have been shown to be an accurate approach for measuring subcutaneous fat at a given location and measuring total subcutaneous fat from the sum of several skinfold sites^12^. Studies often use the sum-of-skinfold thickness taken from around the waist as a measure of central subcutaneous adiposity to assess the efficacy of anthropometric indices used in clinical practice^13^. Predictive equations have been developed based on combinations of anthropometric measures and approximate interrelationships among subcutaneous fat, visceral fat and whole body density. However, biological variations such as age, sex and body type^14^ make estimations of visceral fat using anthropometric techniques difficult^15^.

3D imaging systems capture detailed and accurate external dimensions and shape characteristics of the human body. Measures acquired by these devices are used to describe, interpret and analyse the external dimensions of the human body for applications that include apparel sizing^16,17^ and epidemiological surveys^9,11,18^, with the potential for clinical evaluation and health monitoring^11,19,20^. The point cloud data these systems produce contain all of the differential geometric properties of the body's surface. These surface features could be used to characterise individuals according to their shape as well as their size, to a higher degree of precision and complexity than existing manual methods^9,17^. Previous studies have investigated the use of 3D imaging to create indices that measure human shape, for example, the health index (HI)^21^, the surface-based body shape index (SBSI)^22^ and a Body Shape Index (ABSI)^23^. Recent studies by Loffler-Wirth et al.^9^ and Pluess et al.^24^ are the most sophisticated of these, demonstrating the use of machine learning techniques to evaluate a large number of different human body measurements. These studies demonstrate that large cohorts of participants can be stratified into distinct body-types based on a higher number of independent parameters. However, all of these studies have a specific definition of shape, which is based on the ratios and relative proportions of one-dimensional anthropometrics, such as waist girth and stature. This approach discards the majority of information captured by 3D imaging systems. Therefore, there is a need to investigate more sophisticated methods of analysis that captures all of the complex curvature of the human body and improve current understanding regarding variations in external human form and associated health risk^2,9^.

Humans intuitively perceive differences in body shape between individuals by identifying scale-invariant features, such as surface curvature, body proportions and lateral contours^25^. Recent studies have analysed human body shape using surface curvature derived from 3D scan data to identify differences between individuals and predict body fat percentage^19,25^. These studies stated that further research was needed to associate configurations of these features with distinct body shapes at various scales and to establish reliable associations between body shape and body composition. Geometric morphometrics (GM) is an established method within the fields of anthropology and evolutionary biology to analyse variations in shape. These methods have emerged from established statistical shape theory^26^ and a conceptual understanding of mathematical shape, defined as “what is left when the differences which can be attributed to translations, rotations, and dilations have been quotiented out”^27^ (p. 82). Therefore, to analyse human body shape according to this definition, the effects of non-shape variation—location, rotation and scale—must be removed, which can be achieved using a Procrustes superimposition procedure^28^. Though these types of methods have been used to analyse shape in a wide range of biological and anthropological studies they have not previously been used to analyse the external form of the human body from an anthropometric perspective. The aim of this study was to investigate whether measures of body shape can complement existing anthropometric techniques in the assessment of external human form and the estimation of subcutaneous central adiposity. The objectives of this study were to: demonstrate the application of an analytical procedure for extracting scale-invariant features of human body shape from 3D scan data; compare traditional manual measures and shape measures when assessing variations in external human form; determine whether shape measures can complement manual anthropometric techniques in estimating sum-of-skinfold thickness around the waist. We hypothesise that scale-invariant measures of body shape will identify additional information compared to size measures regarding variations in external human form and will complement existing anthropometric techniques for estimating subcutaneous central adiposity.

## Methods

### Study design

This study is a cross-sectional, observational cohort study designed to determine the efficacy of a novel analytical procedure for measuring variations in human body shape and estimating subcutaneous central adiposity. The Strengthening the Reporting of Observational Studies in Epidemiology (STROBE) statement for cross-sectional studies was followed in the development of this manuscript^29^.

### Participants

Data analysed in this study consists of manual anthropometric profiles, as defined by the International Society for the Advancement of Kinanthropometry (ISAK)^30^, and torso 3D scan data of 43 male participants (Age 33 ± 12 years). Participants in the study cohort were recruited as part of a University-based health screening study using convenience sampling and consisted of University-level students, as well as members of the general public. Before testing all participants completed an initial screening form and provided written informed consent. Participants were required to be over the age of 18 years and able to stand unaided during manual and 3D scan measurement procedures. Levels and frequency of physical activity were self-reported by participants during the initial screening. All participants stated that they performed at least 150 min of moderate-intensity aerobic physical activity per week, in accordance with WHO guidelines^31^. Participants were required to wear non-compressive form-fitting shorts. The study protocol adheres to the principles laid out in the Declaration of Helsinki. All procedures were approved by Sheffield Hallam University Research Ethics Committee, reference number ER5855905. All human measurement methods were performed in accordance with ISO 7250-1:2017 anthropometric standards^32^ and ISAK guidelines^30^.

### Data acquisition

#### Bony landmark palpation

Each participant had bony anatomical landmark locations, which were required for both manual measurement and 3D scan post-processing procedures, manually palpated and marked with a cross on the skin using a fine-tipped surgical marker (e.g. Viscot 1451). All bony anatomical landmarks marked during the experimental protocol are shown in Table 1.

**Table 1:** Bony anatomical landmarks palpated and marked for manual and 3D scan measurement procedures, defined by ISO^32^**.**

Anatomical landmark
Acromiale
Xiphoid process*
Mesosternale
Iliocristale
Anterior superior iliac spine (ASIS)*
9th Thoracic vertebrae*
Subscapulare
Radiale
Iliospinale

*Landmark required for 3D scan post-processing.

#### Manual measurement

All manual measurements were obtained according to standard ISAK procedures by an accredited anthropometrist (Level 1 or 2) to minimise human error in measurement^30^. Measures of body size collected from participants included: stature, body mass, waist and hip girth. Anthropometric indices of weight status were calculated as follows: BMI = mass/stature^2^; WHR = waist girth/hip girth; WHT.5R = waist girth/height^0.5^. All calculations of anthropometric indices involving height and waist girth are recorded in metres (m). Three skinfold sites in close proximity to the measured waist girth were used as the measure of subcutaneous central adiposity, similar to a recent study by Nevill et al.^13^. The three skinfold sites and their definitions were: (1) the iliac crest: a near-horizontal fold superior to the iliac crest; (2) supraspinale: an oblique fold at approximately 45° at the intersection of a line from the anterior superior iliac spine (ASIS) to the anterior axillary fold and a line from the iliac crest; (3) abdominal: a vertical fold 5 cm lateral to the navel. The stature and mass of each participant was measured using a Leicester height stadiometer (Marsden, UK) and digital weight scales (Conair, UK). All girth and skinfold measures were acquired using a basic anthropometric tape measure (Lufkin Executive Thinline 2 m, W606PM) and Harpenden skinfold caliper (Baty International, UK), respectively. The summary characteristics of participants are presented in Table 2. All manual measurements collected from each participant can be found in Supplementary Table S1 online.

**Table 2:** Summary characteristics of participant manual measurements.

Parameter	Mean (SD)	Min	Max	95% CI
Age (years)	33 (12)	18	62	[29, 36]
Stature (cm)	179.8 (7.2)	165.4	193.5	[177.2, 181.6]
Mass (kg)	82.9 (16.2)	50.9	139.4	[78.1, 87.7]
Waist Girth (cm)	86.06 (10.19)	67.3	116.6	[83.0, 89.1]
Hip Girth (cm)	100.36 (7.3)	82.4	120.4	[98.2, 102.5]
BMI (kg m^−2^)	25.7 (4.2)	17.9	38.3	[24.4, 26.9]
Waist-hip-ratio (WHR)	0.86 (0.07)	0.75	1.04	[0.83, 0.88]
Waist by height^0.5^ (WHT.5R)	0.64 (0.08)	0.52	0.84	[0.62, 0.67]
Iliac Crest skinfold thickness (mm)	17.4 (9.4)	3.9	42.0	[14.6, 20.2]
Supraspinale skinfold thickness (mm)	11.7 (6.6)	3.6	29.6	[9.7, 13.7]
Abdominal skinfold thickness (mm)	22.9 (11.6)	4.3	44.4	[19.4, 26.3]
Sum-of-skinfold thickness (mm)	51.95 (26.33)	11.75	101.6	[44.08, 59.82]

*SD* standard deviation, *95% CI* 95% confidence interval.

#### 3D scan measurement

**Figure 1:**
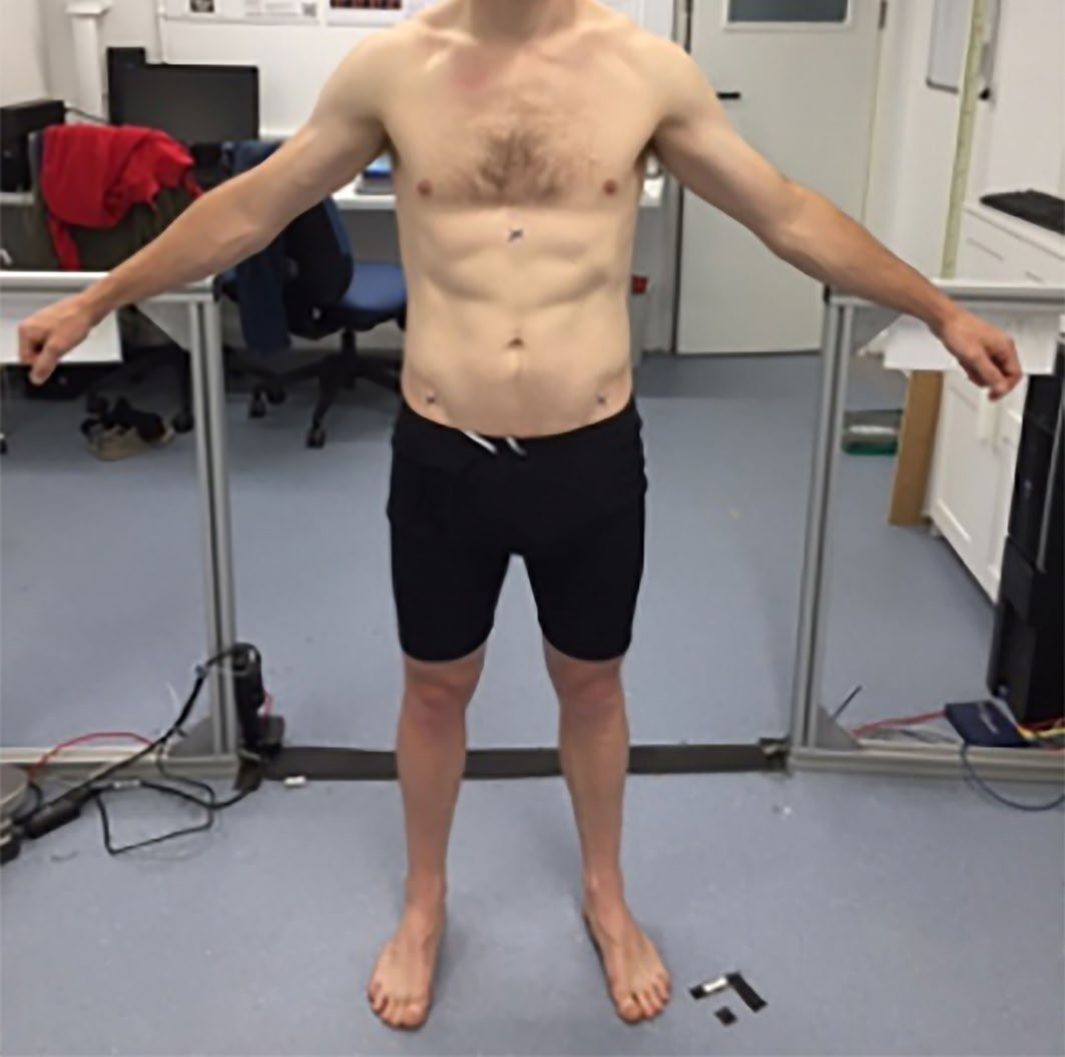
Scanning pose for torso segment scanning adapted from ISO 20685^35^.

3D scan data of the torso was captured using a 3dMD (3Q Technologies Inc., Atlanta, GA) surface imaging system (mean error < 0.5 mm^33^), which has been shown to have acceptable accuracy for acquiring human measurements for clinical studies^34^. This system consists of five synchronised modular units, each containing three machine vision cameras, placed around a square 258 × 258 cm aluminium Bosch (Bosch Rexroth AG) strut frame, using a single computer (64 Bit Windows 7 Professional 4 Core CPU @ 3.6 GHz 8 GB RAM). Calibration and data collection were conducted using 3dMD acquisition software. The calibration procedure followed 3dMD guidelines using a calibration plate and was conducted at the start of each testing session. For torso scanning, participants were asked to adopt a modified version of the standing anatomical pose defined by ISO 20685-1:2018^35^, with their arms abducted by approximately 35◦ (Fig. 1). This ensured that there were no occluded areas of the scan image, whilst enabling participants to maintain a relaxed position during the scanning process. Participants were asked to hold their breath at end-tidal expiration throughout the short scanning duration^36^($\sim$ 15 ms).

### Data analysis

#### 3D scan data post-processing

**Figure 2:**
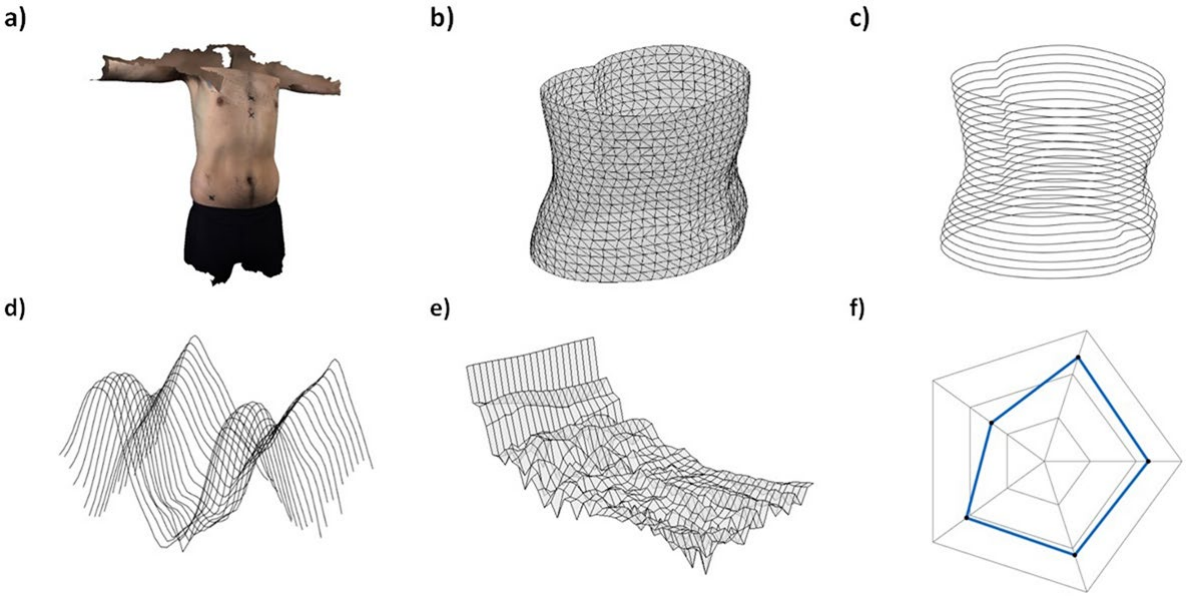
Analytical procedure for extracting shape features from torso 3D scan data; (**a**) Digitise 3D geometry of individual (KinanthroScan v1.0, https://threespace.org/); (**b**) Segment, scale and rotate torso segment; c) Extract transverse data slice profiles; (**d**) obtain signal waveform from profiles; (**e**) Extract frequency content from signals; (**f**) Generate shape features from frequencies.

An experienced researcher (first author) digitised the xiphoid process, ASIS and thoracic vertebra bony landmarks from each 3D scan file (Fig. 2a) using KinAnthroscan—custom software created in-house. The positions of the landmarks were used to create a local anatomical coordinate system located at the centre of each torso scan and according to the convention defined in ISO 20685-1:2018^35^. The centre of the torso was defined as the midpoint between the xiphoid process and the 9th thoracic vertebra. A vector from the xiphoid process to the 9th thoracic vertebra was used as the transverse axis. A vector from the left to the right anterior superior iliac spine (ASIS) markers was used as the sagittal axis. The cross product of these two vectors defined the longitudinal axis. The anatomical axis system enabled differences in translation and orientation between participants in the sample to be removed.

Each torso scan was segmented to include only the coordinate points relating to the region of interest (between the xiphoid process and ASIS markers—Fig. 2b). Twenty-one separate 2 mm transverse data point slices were extracted from each torso segment point cloud at 5% intervals (Fig. 2c). The height of each data slice was set at 2 mm to ensure that the external shape features of the torso segments were preserved while allowing for any gaps in the point cloud. This is based on a previous study by Clarkson et al.^37^, which determined that data slices of 2 mm sufficiently captured the external features of a human torso. However, analysis of other body segments, such as hands or feet, would require greater resolution of data slices. The raw data points within each slice were collapsed to two-dimensions, creating individual shape profiles along the length of the segment. The centroid size (square root of the sum of squared distances of landmarks from the centroid^28^ of all extracted shape profiles within each torso was scaled by a single scale factor so that the sum of distances from each point to the centroid for all slices along the torso segment was equal for all participants. This removed any differences in scale between participants, enabling analysis of body shape according to mathematical shape theory^26^.

#### Dimension reduction

A previous study by Zahn and Roskies^38^, established a method for extracting sets of numerical features from a closed curve that could be used to discriminate between different shapes. It was determined that the coefficients calculated from a fast-Fourier transform can be used to describe the shape features of the original curve^38^. This method was used to extract the Fourier coefficients of the cubic smoothing splines calculated for each data slice profile along the torso segment^39^. The polar coordinates within each data slice, plotted as a continuous signal waveform (Fig. 2d), were inputted to the fast-Fourier transform algorithm in MATLAB (version 9.2, Mathworks, USA) to extract the frequency components present within each data slice (Fig. 2e). Only the first 10 frequency coefficients were used, the higher frequency content was determined to be low amplitude noise. This procedure reduced the total number of variables representing each participant to 210 complex Fourier coefficients.

#### Shape feature detection

Principal components analysis (PCA) was carried out to detect independent features of torso shape that exhibited the highest variation in the sample. The entire list of 42 principal components can be found as Supplementary Table S1 online. This procedure produced 11 principal components that captured 95% of the total body shape variation, resulting in a feature vector to characterise the torso shape characteristics of each participant. The primary shape features capturing the majority of shape variation can then be visualised as a radar diagram (Fig. 2f). 3D scan data post-processing and feature extraction procedures were performed using MATLAB R2018a software (version 9.2, Mathworks, USA).

### Statistical analysis

All body size measures, skinfold measures and derived anthropometric indices were converted into standardised z-scores, ensuring that they were comparable by providing a common scale in units of standard deviations from the mean value of each measure of the cohort. Initially Pearson correlation coefficients were calculated to explore associations between size measures, derived indices and body shape principal components. *P* values < 0.05 were considered statistically significant.

Linear regression analyses were conducted to investigate the strength of associations between surface anthropometrics and sum-of-skinfold measures taken from the waist region of the torso segment. Three different types of regression models were created: (1) Size models, separate regression models for each anthropometric index (BMI, WHR, waist girth and WHT.5R) and a combination of manual size measures (height, mass, waist and hip girth) used as input variables; (2) Shape-only model, a stepwise regression model which used the first 11 torso shape principal components as input variables to determine which contribute to the estimation of skinfold thickness; (3) Combined models, which integrated size measures, anthropometric indices and torso shape principal components as input variables. Each multiple regression model was assessed for multicollinearity between input variables using variance inflation factor (VIF) and tolerance collinearity statistics and for independence of errors using the Durbin-Watson test statistic. If the largest VIF value was greater than 10 there was cause for concern^40^, while tolerance values below 0.2 could indicate potential issues in the model associated with multicollinearity^41^. If the Durbin Watson value differed significantly from 2 this would suggest dependence of errors between input variables in the model^42^. Statistical analyses were performed using SPSS software (version 24.0, IBM, USA).

## Results

### Torso shape features

**Figure 3:**
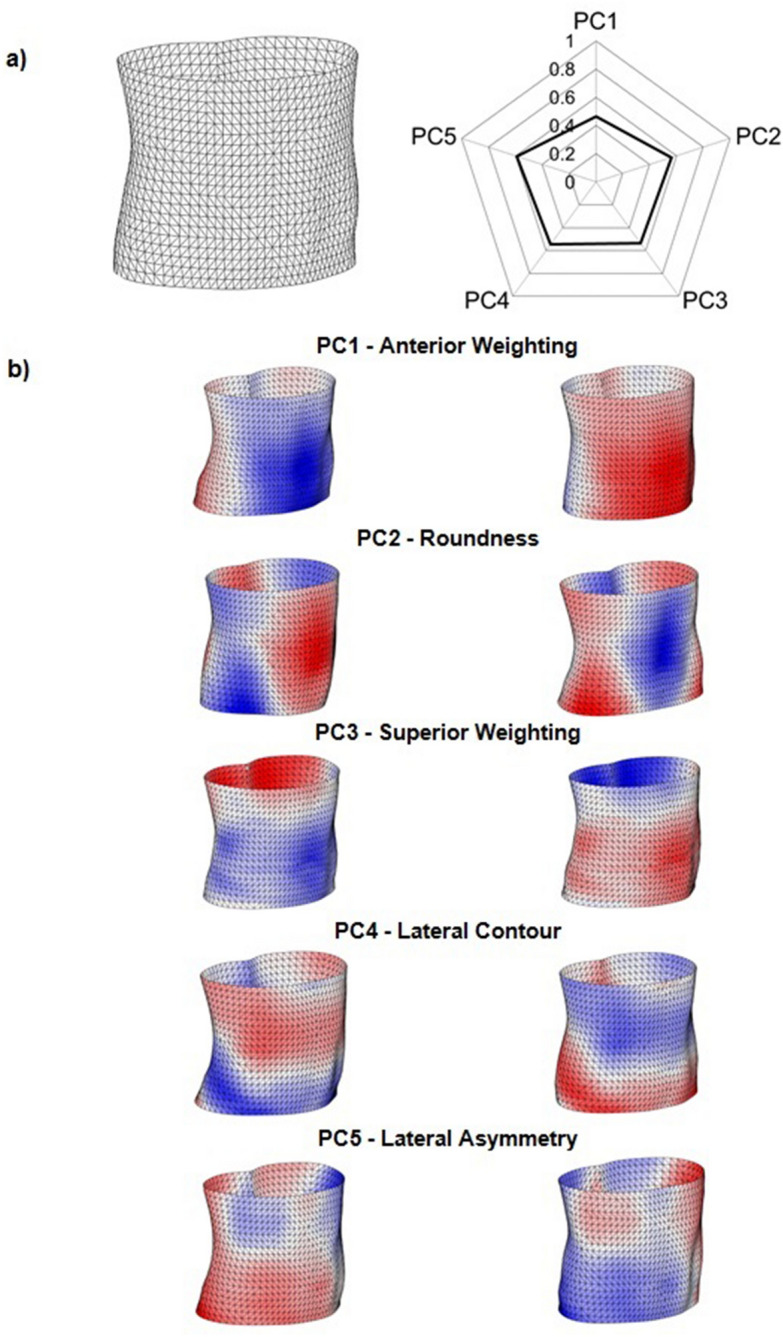
Visualisation of extracted torso shape features; (**a**) Average torso and corresponding radar diagram; (**b**) deviations from the sample mean along the first 5 principal components, the left and right images show the maximum and minimum differences in an individual shape feature from the average torso geometry. Blue and red regions represent areas that protrude less, or more than the average torso, respectively.

Figure 3a shows the meshed surface image of the average torso shape calculated from all participants in the sample. The corresponding radar diagram represents the average values for each of the first 5 principal components identified in this study. Figure 3b shows the maximum and minimum deviations from the average torso shape geometry along each of these first 5 principal components. Blue and red regions represent areas that protrude less, or more than the average torso. The terms used to describe each of the principal components were based on which areas of the torso surface deviated from the average of the sample. For example, PC1—anterior weighting—was based on the observed deviations from the average torso in the anterior and posterior aspect.

### Correlations between size and shape measures

Pearson correlations between size measures, anthropometric indices and shape features are presented in Table 3. Waist girth measure was strongly correlated with hip girth, body mass and derived indices, BMI, WHR and WHT.5R. Hip girth was also strongly correlated with mass, BMI and WHT.5R, though only had weak correlation with WHR. Stature had moderate correlations with body mass and hip girth, but only weak correlations with all other body size and shape measures, suggesting that central adiposity changes independently of body height. PC2 of shape was strongly correlated with waist girth, WHR and WHT.5R, and was also significantly correlated with BMI and other size measures. PC1, PC4, PC5 and PC6 had significant correlations with certain size measures and anthropometric indices, while the remaining shape features were not significantly correlated with size measures. Due to the nature of PCA all extracted shape features were independent of each other and so were uncorrelated. Waist girth and WHT.5R had the strongest correlations with sum-of-skinfold thickness, with several other size and shape measures also significantly correlated with sum-of-skinfold thickness.

**Table 3:** Pearson correlation coefficients between size measures, anthropometric indices and shape principal components**.**

	Size measures				Anthropometric indices			Shape principal components										
	Stature	Mass	Waist Girth	Hip Girth	BMI	WHR	WHT.5R	PC1	PC2	PC3	PC4	PC5	PC6	PC7	PC8	PC9	PC10	PC11
Sum-of-skinfolds	0.02	0.51*	0.72*	0.58*	0.58*	0.55*	0.72*	− 0.30	0.55*	− 0.27	− 0.5*	0.046	− 0.21	− 0.12	0.11	0.14	− 0.07	− 0.09
Stature		0.47*	0.12	0.46*	0.054	− 0.26	− 0.06	0.11	− 0.26	− 0.17	− 0.31	− 0.002	0.27	0.25	0.09	0.18	− 0.02	− 0.25
Mass			0.83*	0.95*	0.90*	0.34*	0.75*	− 0.23	0.48*	− 0.19	− 0.15	0.28	0.40*	− 0.01	0.21	0.18	0.03	− 0.10
Waist Girth				0.79*	0.89*	0.78*	0.98*	− 0.32*	0.76*	− 0.25	− 0.14	0.05	0.08	− 0.01	0.24	0.09	0.06	− 0.15
Hip Girth					0.87*	0.23	0.70*	− 0.26	0.41*	− 0.14	− 0.30	0.37*	0.27*	0.08	0.19	0.11	− 0.03	− 0.07
BMI						0.52*	0.89*	− 0.32	0.67*	− 0.14	− 0.03	0.33*	0.27	− − 0.01	0.23	0.11	0.05	0.02
WHR							0.84*	− 0.24	0.77*	− 0.24	0.063	− 0.28	− 0.14	− 0.31	0.19	0.03	0.10	− 0.18
WHT.5R								− 0.34*	0.81*	− 0.22	− 0.09	0.05	0.03	− 0.19	0.23	0.06	0.07	− 0.11

*P < 0.05

*BMI* body mass index, *WHR* waist-hip ratio; sum-of-skinfolds: Iliac crests skinfold, supraspinale skinfold and abdominal skinfold.

### Regression analysis

Table 4 shows the results of linear regression analyses evaluating the strength of associations between sum-of-skinfold measures taken from the torso segment and each of the commonly used anthropometric indices identified in this study (BMI, WHR, waist girth and WHT.5R). All anthropometric indices significantly improved the prediction of skinfold thickness. WHR had the weakest association, explaining 30.6% of the variance in sum-of-skinfold thickness (R^2^ = 0.306, F(1,35) = 15.434, *p* < 0.01), followed by BMI which explained 33.5% of variance (R^2^ = 0.335, F(1,35) = 17.605, *p* < 0.01). Waist girth (R^2^ = 0.522, F(1,35) = 38.270, *p* < 0.01) and WHT.5R (R^2^ = 0. 522, F(1,35) = 38.258, *p* < 0.01) had the strongest associations, both explaining 52.2% of the variance in sum-of-skinfold thickness measures. Table 5 shows the results of the multiple regression analyses for the size model, the shape model and the combined models. The size-only model, which included all manual body size measures, explained 68.9% of the variance in sum-of-skinfold thickness (R^2^ = 0.689, F(1,32) = 17.690, *p* < 0.01). However, the collinearity statistics suggest that there are serious concerns with this regression model. The tolerance values for mass and hip girth were both below 0.2 and the VIF values for these measures were 17.281 and 11.484, respectively, suggesting high levels of multicollinearity between size measures. In addition, the Durbin-Watson statistic for this model was 2.387, suggesting higher dependence of errors between the input variables. Using stepwise regression for the shape-only model, it was shown that PC2, PC4, PC1 and PC3, in order of their strength of association, contributed significantly to the regression model, explaining 74.2% of the variance in skinfold thickness (R^2^ = 0.742, F(1,32) = 22.96, *p* < 0.01). The Durbin Watson test statistic for this model was close to 2 suggesting independence of errors, and all VIF values for this model were below 10 and all tolerance statistics are above 0.2 suggesting that there was no collinearity between shape features. The results of the combined regression models show that integrating shape principal components with commonly used anthropometric indices improved the estimation of skinfold thickness, with the WHT.5R and shape model explaining 76.5% of the variance in skinfold thickness. The Durbin Watson test statistic for all models was close to 2 suggesting independence of errors for all combined models. All VIF values for all models were below 10 and all tolerance statistics are above 0.2 suggesting that there was not concerning levels of collinearity within the data.

**Table 4:** Linear regression models showing associations between existing anthropometric indices and sum-of-skinfold thickness.

Model	R^2^	Regression equation	Standardised β	F(1,35)	Sig
BMI	0.335	SSF = 0.033 + 0.555*BMI	0.578	17.605	< 0.001
WHR	0.306	SSF = − 0.005 + 0.525*WHR	0.553	15.434	< 0.001
Waist Girth	0.522	SSF = 0.019 + 0.695*Waist	0.723	38.270	< 0.001
WHT.5R	0.522	SSF = 0.02 + 0.694*WHT.5R	0.723	38.258	< 0.001

*SSF* sum-of-skinfolds.

**Table 5:** Multiple linear models showing associations between sum-of-skinfold thickness and (1) size measures; (2) shape PCs; (3) anthropometric indices and shape PCs.

Model	R^2^	Regression equation	DW	Predictor	Standardised β	t	Sig	Collinearity Statistics	
								Tolerance	VIF
Size Measures	0.689	SSF = 0.023 + (0.082*Stature) + (− 1.578*Mass) + (1.067*Waist) + (1.210*Hip)	2.387	Stature	0.084	0.625	0.536	0.534	1.874
				Mass	− 1.640	− 3.999	< 0.001	0.058	17.281
				Waist	1.110	5.125	< 0.001	0.207	4.820
				Hip	1.242	3.716	0.001	0.087	11.484
Shape PCs	0.742	SSF = 0.001 + (0.415*PC2) + (− 0.787*PC4) + (− 0.241*PC1) + (− 0.402*PC3)	2.094	PC2	0.523	5.814	< 0.001	− 0.998	1.002
				PC4	− 0.526	− 5.823	< 0.001	0.991	1.009
				PC1	− 0.350	− 3.866	0.001	0.987	1.014
				PC3	− 0.319	− 3.530	0.001	0.991	1.009
BMI & Shape	0.748	SSF = 0.005 + (0.120*BMI) + (0.350*PC2) + (− 0.778*PC4) + (− 0.213*PC1) + (− 0.376*PC3)	2.164	BMI	0.125	0.918	0.366	0.437	2.290
				PC2	0.441	3.475	0.002	0.504	1.984
				PC4	− 0.520	− 5.729	< 0.001	0.986	1.014
				PC1	− 0.310	− 3.083	0.004	0.803	1.245
				PC3	− 0.298	− 3.198	0.003	0.934	1.071
WHR & Shape	0.743	SSF = − 0.001 + (0.073*WHR) + (0.368*PC2) + (− 0.793*PC4) + (− 0.228*PC1) + (− 0.377*PC3)	1.993	WHR	0.077	0.461	0.648	0.294	3.396
				PC2	0.464	2.966	0.006	0.338	2.960
				PC4	− 0.530	− 5.768	< 0.001	0.981	1.020
				PC1	− 0.331	− 3.318	0.002	0.830	1.205
				PC3	− 0.299	− 2.963	0.006	0.813	1.230
Waist girth & Shape	0.758	SSF = 0.003 + (0.250*Waist) + (0.262*PC2) + (− 0.726*PC4) + (− 0.182*PC1) + (− 0.311*PC3)	2.010	Waist	0.260	1.457	0.155	0.246	4.067
				PC2	0.331	2.082	0.046	0.309	3.233
				PC4	− 0.485	− 5.214	< 0.001	0.902	1.109
				PC1	− 0.265	− 2.492	0.018	0.690	1.448
				PC3	− 0.247	− 2.431	0.021	0.757	1.321
WHT.5R & Shape	0.765	SSF = 0.002 + (0.341*WHT.5R) + (0.192*PC2) + (− 0.731*PC4) + (− 0.158*PC1) + (− 0.291*PC3)	2.006	WHT.5R	0.355	1.745	0.091	0.183	5.450
				PC2	0.242	1.324	0.195	0.227	4.412
				PC4	− 0.488	− 5.416	< 0.001	0.934	1.070
				PC1	− 0.229	− 2.047	0.049	0.607	1.646
				PC3	− 0.231	− 2.283	0.029	0.743	1.345

*SSF* sum-of-skinfolds; *VIF* variance inflation factor, *DW* Durbin-Watson.

## Discussion

It has been suggested that more sophisticated shape indexes, measured using 3D scanning, can surpass manual measures in epidemiology and clinical practice for classifying and health monitoring of individuals^2^. The aim of this study was to investigate whether novel measures of body shape can complement existing anthropometric techniques in the assessment of variations in external human form.

Shape features identified in this study characterise deviations in torso shape that exist within the sample data and are invariant to the effects of scale, location and orientation. The information used to characterise individuals in our study differed from that used in previous studies by Loffler-Wirth et al.^9^ and Pleuss et al.^24^. In these studies, large numbers of simple measures, such as lengths and girths and their ratios, were extracted from 3D body scan data and normalised with respect to height. Machine learning processes were then used to find clusters of participants based on these simple measures. However, though the approach used by Loffler-Wirth et al. identified body types within large cohorts, the primary differences between clusters were variations in the lengths and girths of body segments. In contrast, the approach used in our study has been shown to identify differences in scale-invariant shape features that cannot be captured using traditional anthropometric techniques. There have been recent studies which have also used principal components analysis (PCA) to detect variations in torso 3D scan data similar to our study, such as Ruto et al.^43^ and Ng et al.^44^. However, the torso scan data in both these investigations were not scaled to uniform size, so as a result some variations observed within these studies were related to differences in overall body height and size, as well as variations in scale-invariant body shape. Though the size of the participant samples used in these studies were larger than in our study, the PCA procedure identified the same number of components to describe 95% of the variation present within the cohort. This suggests that shape information inherent within 3D scan data includes subtle variations requiring a greater number of principal components to describe them fully, as opposed to size measures which can be described in a smaller number of components^24^. Though it is currently unknown what all of the shape features captured in this study represents in terms of human health, these results further illustrate the wealth of information regarding body shape and weight distribution which cannot be captured by measurements used in current practice. We have also demonstrated an effective method of capturing and quantifying this information. Given the additional information contained within shape measures an anthropometric procedure that accounts for body shape would be a more effective method of assessing variations in external human form within populations.

External body shape is determined by its skeletal structure and the distributions of fat and muscle mass along its length^1,45^. It has previously been found that the distribution of body fat, especially visceral fat accumulation in the abdominal region, represent the most significant metabolic consequences^7,45,46^. However, the ability of current anthropometric approaches, such as BMI, to determine body fat mass has been questioned repeatedly in previous studies^45,47^. Though the BMI was not originally developed for use specifically as an index of fatness it has been utilised for this purpose because it is a readily obtained metric^45^. However, accumulations of visceral fat do not correlate with total body mass and are therefore not detectable using BMI^14^. Measures such as waist girth and WHT.5R, which utilise measures of body size, have been found to demonstrate improved correlations with quantities of abdominal adiposity and therefore are used as surrogates of central obesity^13^. Regression analyses were conducted to investigate whether torso shape principal components identified in our study contain additional information that can complement these existing anthropometric techniques in the estimation of subcutaneous central adiposity. In this study it has been shown that shape principal components explained 74.2% of the variance in sum-of-skinfold thickness, compared to 52.2% explained by existing anthropometric indices waist girth and WHT.5R. However, when waist girth and WHT.5R were combined with torso shape principal components they were able to explain 75.8% and 76.5% of the variance in sum-of-skinfold thickness, respectively. These results agree with those of Nevill et al.^13^, which found that WHT.5R was the most sensitive of existing anthropometric indices to changes in abdominal adiposity, however, the addition of scale-invariant measures of body shape can improve this prediction still further. These results are promising for a study conducted on a small cohort. Though the addition of greater numbers of predictor variables will always improve the accuracy of a regression model, the torso shape features extracted using our analytical procedure are independent and describe different aspects of human form. This is contrary to individual manual measures of body size (stature, mass, waist and hip girth), which have been shown to exhibit high levels of collinearity, preventing them being from combined in the same regression model. For this reason, anthropometric indices, such as BMI, WHR and WHT.5R, are used as a way to combine measures of body size to create proxies of body shape, reducing the complexity of human form to a single value. However, our study has shown that distinct features of body shape can be measured directly, providing additional information that can be used to complement existing anthropometric techniques in the estimation of central adiposity. Though it is acknowledged that current anthropometric proxies of visceral adiposity, such as waist girth and WHT.5R, are confounded by levels of subcutaneous fat^15,48^, the additional information contained within shape measures may be able to identify features of external human form that relate to accumulations of visceral fat and associated cardio-metabolic health risks. Further study is required to establish these relationships, as well as the effects on shape measurement caused by underlying health issues, such as edema, which could obscure relationships between shape and body composition.

A limitation of this study was the restricted size of the participant cohort; it does not capture the complete range of body shapes that exist in the wider population and may limit the effectiveness of PCA used to detect features of torso shape variation in this study. In order to be robust, methods of 3D body classification require several thousand participants^9^. Therefore, the next stage of work will be to apply our analytical procedure to the Leipzig Research Centre for Civilization Diseases (LIFE) dataset, one of the world’s largest collections of 3D body scan data with over 10,000 participants^49^. The increased size of this cohort will enable a greater range of body shapes and sizes to be characterised and add further stability to the results of the PCA. The LIFE dataset is also supplemented with medical examination results, such as MRI scans of visceral adipose tissue volume and oral glucose tolerance tests (OGTT), which could be used to further investigate relationships between body shape and cardio-metabolic risk factors. The long-term aim of this work will be to combine shape parameters identified within this large dataset with traditional size anthropometrics to improve body composition predictive power and the quality of health classification.

## Conclusions

This paper introduces a novel method for extracting features which characterise an individual’s body shape. This characterisation of shape contains information that is absent from measures used in current anthropometric practice. In addition, these identified shape features can complement traditional anthropometrics when explaining variations in quantities of subcutaneous abdominal adiposity. The aim of future work will be to apply the proposed methods to characterise a large cohort of several thousand participants and identify patterns of variation across a wider range of body shapes and to further investigate the relationship between shape features and physical health indicators.

## Supplementary information

Supplementary information

## Data Availability

All data generated or analysed during this study are included in this published article (and its Supplementary Information files).
